# Sporadic MM-1 Type Creutzfeldt-Jakob Disease With Hemiballic Presentation and No Cognitive Impairment Until Death: How New NCJDRSU Diagnostic Criteria May Allow Early Diagnosis

**DOI:** 10.3389/fneur.2018.00739

**Published:** 2018-09-05

**Authors:** Lorenzo Saraceno, Vito A. G. Ricigliano, Michele Cavalli, Alessandro Cagol, Giovanna Bosco, Fabio Moda, Paola Caroppo, Giovanni Meola

**Affiliations:** ^1^Department of Biomedical Sciences for Health, University of Milan, IRCCS Policlinico San Donato, Milan, Italy; ^2^Department of Neurology, IRCCS Policlinico San Donato, Milan, Italy; ^3^Carlo Besta Neurological Institute, IRCCS Foundation, Milan, Italy

**Keywords:** Creutzfeldt-Jakob disease, prion disorders, hemiballic syndrome, cognitive impairment, NCJDRSU, CJD diagnostic criteria, methionine/methionine polymorphism

## Abstract

Sporadic Creutzfeldt-Jakob disease is the most common human prion disorder. Although associated with heterogeneous clinical phenotypes, its distinctive feature is the presence of a rapidly progressive multidomain cognitive impairment. We describe the atypical case of a patient affected by sporadic Methionine/Methionine type 1 Creutzfeldt-Jakob disease (typically associated with early cognitive decline) who presented with an isolated hemiballic syndrome and no signs of cognitive involvement until death. We review sporadic Creutzfeldt-Jakob disease diagnostic criteria and their updates since their first formulation, highlighting their limitations in clinical diagnostic work-up. Finally, we discuss the recently introduced National Creutzfeldt-Jakob Disease Research and Surveillance Unit diagnostic criteria, suggesting how their application could support an early clinical diagnosis, even in atypical cases, such as the one presented.

## Background

Prion disorders are a group of conditions that affect the central nervous system, progressively impairing cognitive and motor functions. Among human prion diseases, sporadic Creutzfeldt-Jakob disease (sCJD) is the most common one, accounting for 85–90% of cases, and is often rapidly progressive with early psychiatric symptoms and pervasive cognitive decline (1). Neuropsychological impairment is observed at disease presentation or in advanced phases in almost 100% of subtypes associated with a specific Methionine/Methionine (MM) polymorphism in codon 129 of the prion protein (PrP) gene (2). We report a case of MM-1 type sCJD with very atypical clinical onset characterized by isolated hemiballic syndrome and cognition sparing until death. In such condition, given the presence of an intact cognitive profile, sCJD diagnosis is unfeasible according to revised WHO (3) as well as European MRI-CJD Consortium (4) and UCSF (5) criteria. However, the recent development of novel ultrasensitive seeding assays (Real-time quaking-induced conversion) and the updated National CJD Research and Surveillance Unit (NCJDRSU) diagnostic criteria (6, 7)^1^ opened up a new scenario that may allow early sCJD diagnosis even in atypical clinical presentations.

## Case presentation

In December 2015 a previously healthy 61-year-old woman started complaining of slowly progressing unsteadiness of gait due to left limb coordination impairment. In January 2016 she was referred to our Emergency Department for the subacute onset of unintentional sharp movements of left limbs, initially causing repeated falls and, later on, impossibility to reach and maintain the standing position. Neurological examination showed left limb proximal hyperkinetic-hemiballic movements with mild distal dystonic posture, mild asymmetric left-sided plastic rigidity and ipsilateral pyramidal signs. Cognitive function was normal (Mini-Mental Status Examination, MMSE 30/30), without behavioral, language, or psychiatric abnormalities.

Laboratory tests were in range except for severe hypercalcemia (15.1 mg/dl) related to primary hyperparathyroidism (PTH 422 pg/ml), which was gradually corrected with oral administration of cinacalcet, intravenous hydration, and zoledronic acid, without any improvement of signs and symptoms.

**Figure 1:**
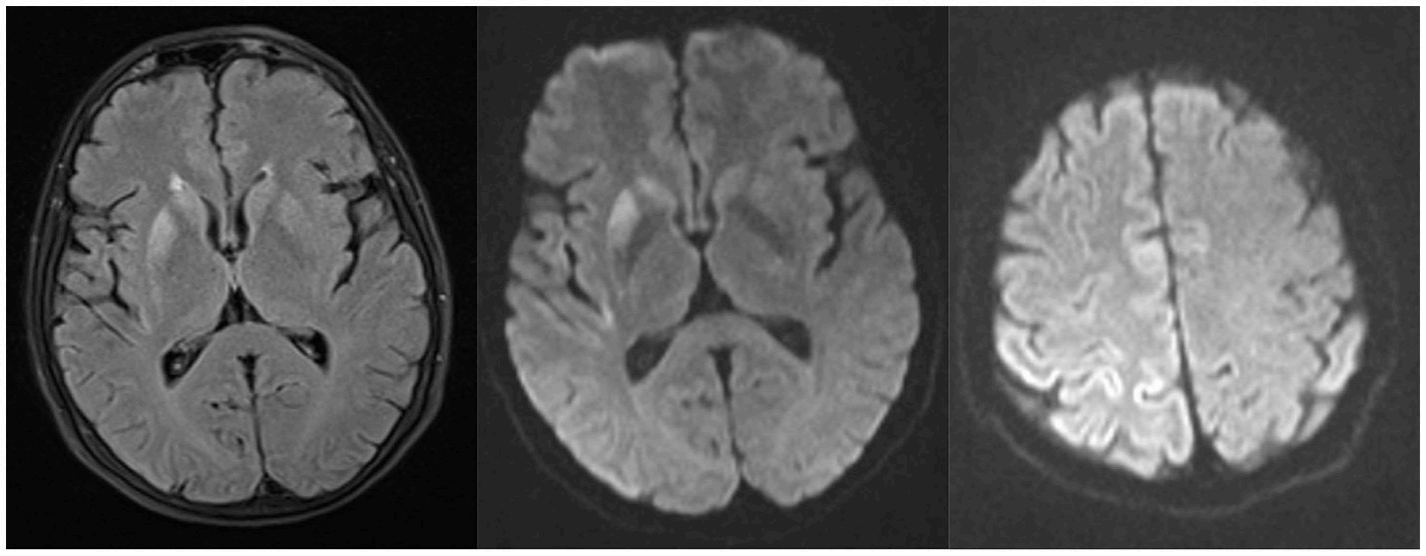
Brain MRI showing faint hyperintensity on Fluid Attenuated Inversion Recovery (FLAIR) images and positive diffusion-weighted (DWI) signal in the right lenticular and caudate nuclei, posterior insular and fronto-parietal cortex, without cortical atrophy or gadolinum enhancement.

Brain computed tomography (CT) scan was normal, while brain magnetic resonance imaging (MRI) showed faint hyperintensity on Fluid Attenuated Inversion Recovery (FLAIR) images and positive diffusion-weighted (DWI) signal in the right lenticular and caudate nuclei, posterior insular and fronto-parietal cortex, without cortical atrophy or gadolinum enhancement (Figure 1). Total body CT scan with administration of iodinated contrast was unremarkable.

**Figure 2:**
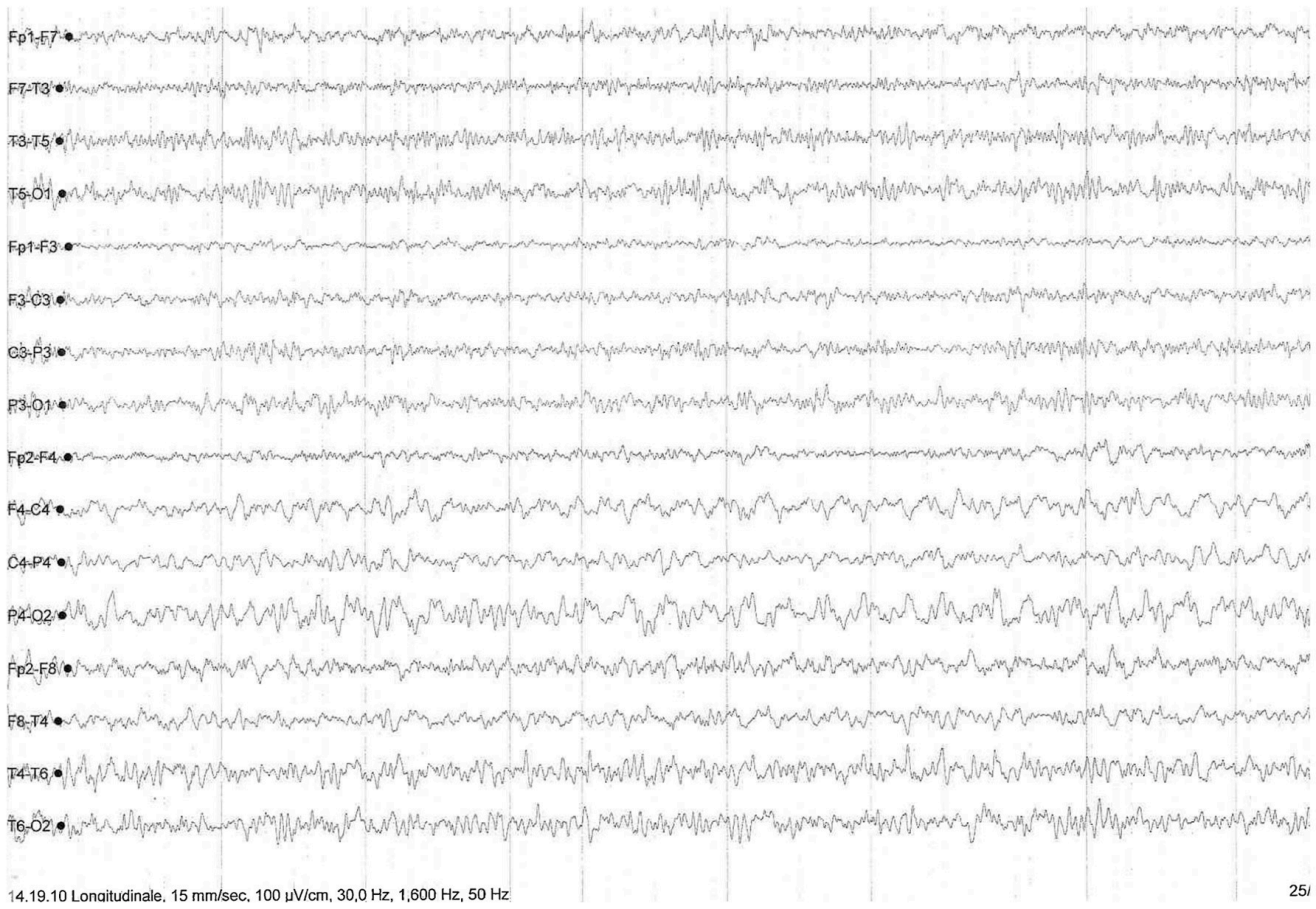
EEG showing nonperiodic and right-prevailing abnormal slow waves in fronto-temporal-parietal regions.

Autoimmune screening, tumor markers, ceruloplasmin with seric and urinary copper dosage, paraneoplastic antibodies (Hu, Yo, Ri, Ma1-2, CV2/CRMP5, amphiphysin, GAD) on both blood and cerebrospinal fluid (CSF) were performed, with negative results. CSF screening for fungal, bacterial or viral infections, as well as Rickettsiosis, Borreliosis and HTLV1-2 infection (performed because of the patient's history of an insect bite during a trip in Madagascar 2 weeks before symptoms onset), yielded negative results. Despite her normal cognitive profile confirmed at 3 months after clincal onset, on the basis of clinical manifestations and brain MRI images, a prion disorder was suspected. CSF Tau protein was significantly increased (2229 pg/ml; normal values 51–70) and 14.3.3 protein was weakly positive, with normal P-tau and β-amyloid values, thus confirming the hypothesis of a neurodegenerative process. Notably, repeated electroencephalography (EEG) showed nonperiodic right-prevailing abnormal slow waves in fronto-temporal-parietal regions (Figure 2) without typical periodic triphasic sharp waves. DNA sequencing for PrP gene mutations of inherited CJD on blood samples showed no alterations, while analysis of codon 129 detected a MM polymorphism.

**Figure 3:**
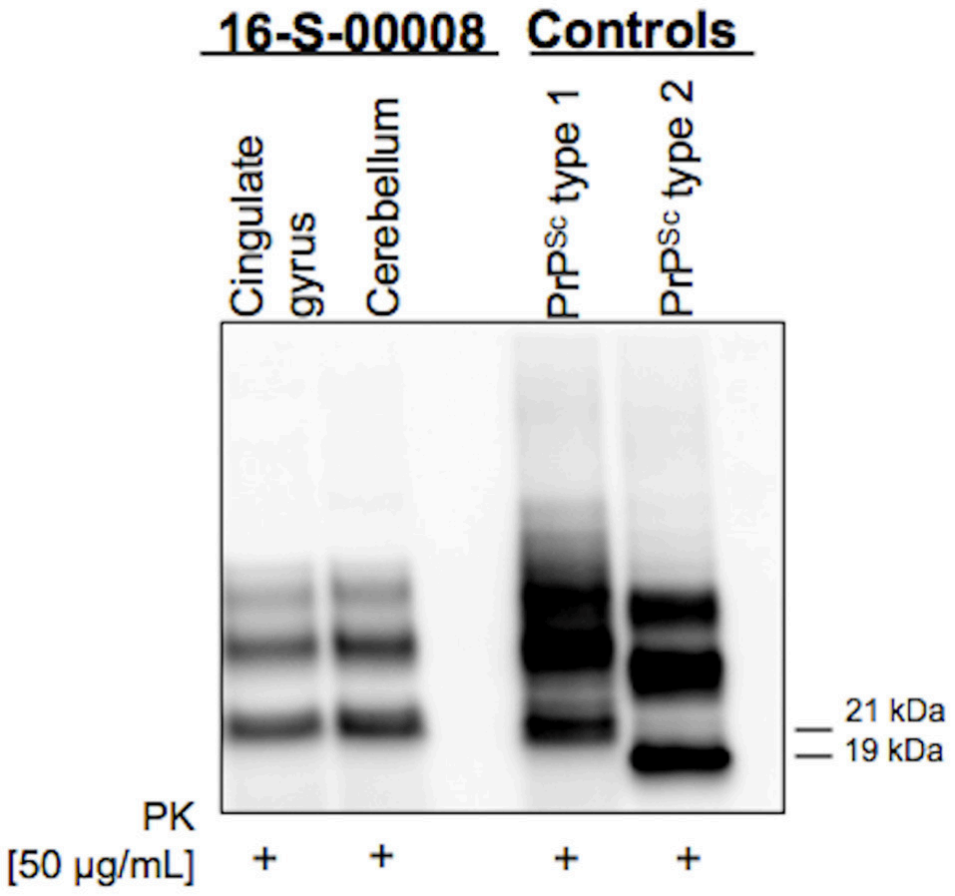
Western Blot showing type 1 abnormal isoform of the prion protein (PrPSc) in both cingulate gyrus and cerebellum brain samples.

In the following weeks, the patient experimented rapid worsening of symptoms, with neck dystonia, diffuse hypertonic rigidity, startle reaction, myoclonus at rest on the left limbs, dysarthria, hypophonia, dysphagia, and inappetence. Myoclonus polygraphic recording was not performed in the clinical workup since neurophysiologic data would not have modified the degree of diagnostic certainty according to current criteria (3–6)^1^. A second brain MRI was repeated, resulting unchanged. Clonazepam oral drops 2.5 mg/mL were started, with benefit only on myoclonic jerks. To note, a specific neuropsychological (NPS) panel including MMSE, Frontal Assessment Battery (FAB), Clock Drawing Test (CDT), Digit Span (DS), Corsi Block-Tapping Test (CBTT), Story Recall Test (SRT), Trail Making Test (TMT), Attentional Matrices (AM), Controlled Oral Word Association Test (COWAT), Semantic Fluency Test (SFT), Arm Ideomotor Apraxia Test and Rey-Osterrieth Complex Figure Test (ROCF), performed in March 2016 was substantially within the range of normative scores (Table 1). Due to poor clinical conditions and concurrent pneumonia, the patient died in April 2016, 4 months after disease onset. Post-mortem immunoblotting for PrP protein on nervous tissue sampled from right brain emisphere confirmed the diagnosis of sCJD, by the detection of type 1 abnormal isoform of the prion protein (Figure 3). To note, immunohistochemistry and histology were not performed since fixed brain tissue samples were not available.

**Table 1:** Neuropsychological (NPS) panel.

**NPS panel**	**Raw score**	**Adjusted score**	Equivalent score^*^
Mini Mental Status Examination (MMSE)	30/30	30/30	–
Frontal Assessment Battery (FAB)	16	15.5	3
Clock Drawing Test (CDT)	1/6	–	–
Digit Span (DS)	6	5.75	4
Corsi Block-Tapping Test (CBTT)	5	5	4
Story Recall Test (SRT)	11	10	3
Trail Making Test (TMT)- Part A	45	36	4
Trail Making Test (TMT)- Part B	120	94	4
Attentional Matrices (AM)	47	44.5	3
Controlled Oral Word Association Test (COWAT)	34	32.5	4
Semantic Fluency Test (SFT)—Animals	17	17.5	4
Arm Ideomotor Apraxia Test—Upper right limb	69	68	3
Arm Ideomotor Apraxia Test—Upper center limb	66	65	2
Rey-Osterrieth Complex Figure Test (ROCF)—Copy	29	29.25	1
Rey-Osterrieth Complex Figure Test (ROCF)—Recall	10	12.5	2

* *Equivalent Scores (ES) is a 5-point scale that offers a solution to the problem of standardizing neuropsychological scores after adjustment for age and education. ES = 0 corresponds to pathological performance, E = 1 to a borderline performance, E ≥2 to a normal performance (7)*.

## Discussion

The case of sCJD documented above has some unusual and unreported features. First, disease onset was characterized by the atypical subacute presentation of a left hemiballic syndrome that worsened over time but remained the unique isolated clinical feature for about 1 month.

More in detail, although extrapyramidal movement disorders—such as rigidity, tremor, alien limb, hypo/akinesia, dystonia, athethosis, choreoathetosis, tremor, hemiballismus, and myoclonus—are frequently described during disease course (8, 9), their specific presentation as isolated syndrome at disease onset is very rare. Notably, few articles have been published reporting CJD clinical onset with myoclonus, tremor and focal, or unilateral dystonic postures (9), isolated choreoathetosis (10), but, to our knowledge, no cases of CJD starting with prominent hemiballic features have been described so far. In our patient symptoms of left limb incoordination rapidly outbroke, in a 4-week course, into sudden and flinging left-sided disabling proximal involuntary movements.

The second atypical feature of our report was the absence of cognitive impairment. Indeed, cognitive profile in sCJD, when systematically evaluated by specific NPS tests, usually shows a multidomain impairment, with evidence, since early stages of disease when global MMSE is 30/30, of a dysexecutive syndrome as the leading cognitive symptom, associated with prominently expressive speech disorder and a plethora of parietal signs, including visuospatial impairment and apraxia (2). Nonetheless, in our patient NPS panel performed at the third month of her 4-month disease history showed no cognitive impairment, except for a mildly pathological performance in Rey figure copy, suggesting only minimal visual-perceptual deficit (Table 1). In addition, no behavioral or psychiatric abnormalities were detected during 1-month hospitalization in our Department, nor were reported by her relatives until death, which occurred 4 months after symptom onset and 1 month after discharge from our Department. To note, even though other unrelated causes (specifically, pneumonia) might have contributed to accelerate death, still it is indisputable the disproportion between cognitive integrity at her neuropsychological evaluation and advanced motor extrapyramidal deficits that caused complete loss of autonomy and confined the patient to bed already at the second month after disease onset (Barthel Index scale 15/100; Activities of daily living scale 1/6, Instrumental Activities of daily living 0/8).

Indeed, such discrepancy has been rarely observed in clinical practice. This phenotype could be partly explained by a disproportionate impairment of basal ganglia with respect to cerebral cortex, as confirmed by brain MRI, which showed prevalent DWI hyperintensity of the right lenticular and caudate nuclei with only mild cortical involvement and no atrophy.

The identification of a MM codon 129 polymorphism of the PrP gene in the absence of pathological mutations of the protein, together with post-mortem immunoblotting, led us to define the case as MM-1 type sCJD (Figure 3). This datum strengthens the peculiarity of our report, since it is known that, according to the current classification of human prion disorders (11), MM subtypes (M1 and M2) are traditionally considered cognitive variants, typically presenting with early and rapidly progressing multidomain cognitive impairment (12, 13). Furthermore, based on clinical most commonly used diagnostic criteria (Table 2), a persistently unaffected cognitive profile, even in the presence of typical MRI alterations, does not even allow to reach the diagnostic level of “*possible*” sCJD.

**Table 2:** Sporadic Creutzfeldt-Jakob disease (sCJD) diagnostic criteria.

**World Health organization (WHO) criteria, 1998 (3)**	**University of California, San Francisco (UCSF) criteria, 2007 (4)**	**European MRI-CJD Consortium criteria, 2009 (5)**	**National CJD research and surveillance unit (NCJDRSU) diagnostic criteria, 2017 (6)^1^**
**DEFINITE:** -**1.1** I + 2 of II and Neuropathologically or immunocytochemically or biochemically confirmed **PROBABLE:** - **1.2** I +2 of II and 1 of III **POSSIBLE:** -**1.3** I + 2 of II + duration < 2 years	**DEFINITE:** - **1.1** I + 2 of II and Neuropathologically or immunocytochemically or biochemically confirmed **PROBABLE:** - I +2 of II and 1 of III **POSSIBLE:** - not defined in this criteria set	**DEFINITE:** - **1.1** I + 2 of II and Neuropathologically or immunocytochemically or biochemically confirmed **PROBABLE:** - I +2 of II and 1 of III **POSSIBLE:** - **1.3** I + 2 of II + duration < 2 years	**DEFINITE:** - **1.1** Progressive neurological syndrome AND Neuropathologically or immunocytochemically or biochemically confirmed **PROBABLE:** - **1.2.1** I + 2 of II and 1 of III (EEG ^*a^) *OR* - **1.2.2** I + 2 of II and 1 of III (MRI^*3^) *OR* - **1.2.3** I + 2 of II and 1 of III (positive 14-3-3) *OR* - **1.2.4** Progressive neurological syndrome and positive RT-QuIC in CSF or other tissues **POSSIBLE:** - **1.3** I + 2 of II + duration < 2 years
I.Progressive dementia	I. Rapid cognitive decline	I. Progressive dementia^*^	I. Rapidly progressive cognitive impairment
II. Two among: - Myoclonus - Piramidal/extrapiramidal symptoms - Visual/cerebellar dysfunction - Akinetic mutism	II. Two among: - Myoclonus - Piramidal/extrapiramidal symptoms - Visual Impairment - Cerebellar dysfunction - Akinetic mutism - Focal cortical signs (e.g. neglect, aphasia, acalculia, apraxia	II. Two among: - Myoclonus - Piramidal/extrapiramidal symptoms - Visual/cerebellar dysfunction - Akinetic mutism	II. Two among: - Myoclonus - Visual or cerebellar problems - Piramidal or extrapiramidal features - Akinetic mutism
III. One among: - Typical EEG ^*a^ *OR* - Elevated CSF 14.3.3 protein (total disease duration < 2y)	III. One among: - Typical EEG ^*b^ *AND/OR* - Typical MRI ^*1^	III. One among: - Typical EEG ^*a^ *OR* - Elevated CSF 14.3.3 protein (total disease duration < 2y) *OR* - Typical MRI^*2^	III. One among: - Typical EEG ^*a^ *OR* - Elevated CSF 14.3.3 protein *OR* - Typical MRI ^*3^ *OR* - positive real-time quaking-induced conversion in CSF or other tissues

*a *Generalized periodic complexes*.

*b *Periodic latelalized epileptiform discharges (PLEDs)*.

*1 *Subcortical or cortical gyral hyperintensity (cortical ribboning) on DWI and preferably restricted diffusion on ADC map*.

*2 *High-signal intensity on either FLAIR or DWI in both the putamen and the caudate nucleus or in at least two cerebral cortical regions (from either the temporal, occipital, or parietal cortices, not including frontal or limbic regions)*.

*3 *High signal in caudate/putamen on MRI brain scan or at least two cortical regions (temporal, parietal, occipital) either on DWI or FLAIR*.

* *European MRI-CJD Consortium criteria (2009) clinical criteria are equal to those included in World Health organization criteria (1998). However, due to a typographical error in the Figure 1 of Zerr article (13), progressive dementia was not required in the clinical criteria and dementia was wrongly incorporated as a clinical symptom instead of myoclonus (1)*.

Indeed, according to commonly used diagnostic criteria, “*possible*” diagnosis is achievable only in presence of the classical clinical phenotype characterized by (1) rapidly progressive (< 2 years) dementia plus (2) two elements among (a) myoclonus, (b) cerebellar syndrome or visual dysfunction, (c) pyramidal or extrapyramidal signs, and (d) akinetic mutism (1, 3, 4, 5).

Despite diagnostic criteria recurrent updates, “*definite*” sCJD diagnosis is actually still dependent on the neuropathologic exam and the subsequent scrapie prion protein identification by immunochemistry or, as performed in our case, by Western blot (3, 4, 5). Starting from this point, the clinical need for an earlier diagnosis led, in the last 40 years, to the identification of multiple ancillary instrumental tests which could help obtain a “*probable*” sCJD diagnosis, in presence of the typical clinical portrait described above.

Thanks to the possibility to detect periodic sharp-waves complexes (PSWC) in about two-thirds of patients, usually in the late stage of the disease, EEG has been since 1979 the first paraclinical auxiliary investigation commonly used in clinical practice (1, 4, 14).

The second investigation introduced was CSF Western blot detection of the 14.3.3 protein, a non prion-specific biomarker of acute neuronal injury. The clinical usefulness is however controversial because of a variable diagnostic sensitivity and specificity, respectively ranging from 53 to 97% and from 40 to 100% (1, 6).

Nevertheless, together with EEG, 14.3.3 protein detection was included in the 1998 World Health Organization diagnostic classification criteria as an auxiliary exam to support a “*probable*” sCJD diagnosis (3). A recent study by Peckeu et al., based on 1572 autopsied patients in France, showed how the introduction of 14.3.3 detection in the definition of probable cases has provided an increase of diagnostic sensitivity from 56.1 to 82.4%, but a loss of specificity from 95.6 to 75.6% (6).

In order to overcome diagnostic WHO criteria limitations, developed for epidemiologic surveillance purposes rather than for early diagnosis (1), in 2009 and in 2011 the European MRI-CJD Consortium (4) and the UCSF (5) criteria were respectively elaborated.

In these updates, revising 1998 WHO criteria, MRI data were introduced as equivalent to elevated 14.3.3 proteins or PSWC to identify “*probable*” sCJD. FLAIR or DWI hyperintensity in both putamen and the caudate nucleus or in at least two cortical regions from either the temporal, occipital or parietal lobes (Table 2) showed to have alone a sensitivity and specificity of 83% (4). A further step toward an early sCJD diagnosis has recently been made with the development of novel ultrasensitive seeding assays (Real-time quaking-induced conversion, RT-QuIC) that directly detect the amplified pathological prion protein in the CSF or in the olfactory mucosa with very high sensitivity and specificity (respectively 96 and 100%) (15, 16). RT-QuIC test, not performed on our patient since in 2016 it was available only for research purposes, could help in the future in obtaining a definitive antemortem sCJD diagnosis and in January 2017 has been integrated in the updated NCJDRSU diagnostic criteria for human prion disease (Table 2) (6)^1^. To note, NCJDRSU has not merely introduced RT-QuIC test positivity in CSF or other tissues to reach the diagnostic level of “*probable”* sCJD, as done at the time of the introduction of EEG, MRI and CSF 14.3.3 protein detection in criteria revisions. Indeed, the experts have established (1.2.4 criterion) that, due to its high specificity, RT-QuiC, if combined with a “*progressive neurological syndrome*,” allows a *probable*” sCJD diagnosis without the need of a concurrent cognitive decline or dementia^1^. In conclusion, in case of atypical onset and absence of rapidly progressive cognitive impairment, according to the diagnostic criteria most commonly used to date in clinical practice (3, 4, 5), an early sCJD diagnosis is not possible. However, the new NCJDRSU diagnostic criteria may provide an early diagnosis of “*probable sCJD*,” also in unusual cases like the one described.

## Ethics statement

Diagnostic work-up and case report description were conducted according to the principles expressed in the Declaration of Helsinki, the institutional regulation and Italian laws and guidelines. Written informed consent for the publication of this case report was obtained from the patient's husband after her death.

## Author contributions

LS wrote the manuscript. LS, VR, MC, and AC made table and figures. LS, VR, MC, AC, and GM reviewed the literature. GB performed neuropsychologial evaluations. LS, VR, MC, AC, and GM performed final manuscript review and editing. FM and PC performed western blot, interpreted results and provided its picture.

### Conflict of interest statement

The authors declare that the research was conducted in the absence of any commercial or financial relationships that could be construed as a potential conflict of interest.

## Footnotes

^1^https://www.cjd.ed.ac.uk/sites/default/files/diagnostic%20criteria.pdf
